# Young children’s development after forced displacement: a systematic review

**DOI:** 10.1186/s13034-024-00711-5

**Published:** 2024-02-01

**Authors:** Katharina Bernhardt, Saskia Le Beherec, Jana R. Uppendahl, Melia Fleischmann, Matthias Klosinski, Luisa M. Rivera, Georgia Samaras, Martha Kenney, Ruth Müller, Ina Nehring, Volker Mall, Andrea Hahnefeld

**Affiliations:** 1 Chair of Social Pediatrics, TUM School of Medicine, Technical University of Munich, Munich, Germany; 2 Kbo Kinderzentrum, Heiglhofstrasse 65, 81377 Munich, Germany; 3 Department of Anthropology, Emory University, Atlanta, GA USA; 4 Department of Science, Technology and Society, Technical University of Munich, Munich, Germany; 5 Department of Women and Gender Studies, San Francisco State University, San Francisco, CA USA; 6 School of Management, Technical University of Munich, Munich, Germany; 7 School of Life Sciences, Technical University of Munich, Munich, Germany

**Keywords:** Refugee, Displacement, Child, Preschool, Social-emotional development, Cognitive development

## Abstract

**Objectives:**

To examine the impact of displacement experiences on 0- to 6-year-old children’s social-emotional and cognitive development, as well as influencing factors on reported outcomes.

**Study design:**

We systematically searched MEDline, Psyndex, Cochrane Library, Web of Science, Elsevier, TandF, Oxford Journal of Refugee Studies, Journal of Immigrant & Refugee Studies, and Canada’s Journal on Refugees for existing literature regarding social-emotional and cognitive outcomes in children directly exposed to forced displacement due to political violence. Results were synthesized in the discussion and displayed using harvest plots.

**Results:**

Our search generated 9,791 articles of which 32 were selected for review and evaluation according to NICE criteria. Included studies provided results for 6,878 forcibly displaced children. Measured outcomes were diverse and included areas such as peer relations, prosocial behavior, family functioning, play, intelligence, learning performance, and language development. Repeated exposure to adverse experiences, separation from parents, parental distress, as well as duration and quality of resettlement in the host country were reported as influencing factors in the reviewed studies.

**Conclusion:**

As protective factors like secure and stable living conditions help to promote children’s development, we call for policies that enhance participation in the welcoming society for refugee families. Early integration with low-threshold access to health and educational facilities can help to mitigate the wide-ranging negative consequences of forced displacement on young children’s development.

**Supplementary Information:**

The online version contains supplementary material available at 10.1186/s13034-024-00711-5.

## Introduction

Adverse childhood experiences (ACE) are associated with an increased risk for disadvantageous developmental trajectories [1, 2]. The first years in life especially are a critical time period characterized by rapid physiological, cognitive and emotional changes [3, 4]. During this time, learning experiences are required for children to reach important developmental milestones in areas such as language acquisition, executive functions, perspective taking, emotion regulation, or social interaction [5]. When children are hindered from making these essential experiences within specific timeframes, they may encounter challenges in social-emotional and cognitive development—therefore placing exposed infants at high risk for poor outcomes in the long-term [2]. Displaced children are a particularly vulnerable population at high risk for exposure to ACEs before, during and after flight [6] (See *Results: Adverse childhood experiences* for a detailed definition). In addition, their everyday lives in refugee accommodations are often characterized by insecurity, frequent changes in housing, limited access to school and health institutions, and a lack of interaction with peers or play material, which further restricts them from a stable, age-appropriate environment in a developmentally important phase of life [7]. Young infants are highly dependent on their caregivers [8]. Although displaced parents are committed to providing a secure and caring environment for their children, even in uncertain circumstances, they may face constraints due to their own exposure to adversities or structural inequalities, which further jeopardizes the child's development [9].

More than 43 million minors were forcibly displaced worldwide in 2022 [10]. In Germany, 20.4% of asylum applications were filed for children under the age of six, making them one of the largest groups among German asylum seekers [11]. While the negative consequences of forced displacement and associated risk factors on older children’s and adolescents’ mental health and development are well documented [7, 12–18], only one systematic review has outlined the effects of forced displacement on children of preschool age [19]. While this work has highlighted high rates of posttraumatic stress disorder (PTSD), sleep problems, disturbed play, and somatic complaints among young, displaced children, to our knowledge, no such work exists on developmental outcomes in this population. Understanding the effects of forced displacement on young children’s progression is important, as developmental difficulties are considered predictors of later health and academic problems [13].

Our systematic review aims to address this gap by (a) capturing existing literature on markers of social-emotional and cognitive development in forcibly displaced children 0- to 6-years of age and (b) reviewing influencing factors associated with these outcomes.

## Methods

### PRISMA

Preparation and reporting of evidence in this systematic review is based on the Preferred Items for Systematic Reviews and Meta-analysis (PRISMA) reporting guidelines [20].

### Search strategy

The following databases were searched from May 2021 until October 2023: MEDline, Psyndex, Cochrane Library, Web of Science. Additionally, libraries of the publishers Elsevier and TandF, the Oxford Journal of Refugee Studies, Journal of Immigrant & Refugee Studies and Canada’s Journal on Refugees and reference sections of related systematic reviews were hand-searched for eligible articles. Results were found from 1940 to 2023. We included a wide range of search words regarding possible effects on the developmental outcomes of displaced children: (refugee OR flight OR resettle* OR displace* OR migrat* OR asylum seeker) AND (child* OR preschool* OR kindergarten) AND (social OR emotion* OR peer OR relation OR behavior OR behaviour OR intelligence OR iq OR memory OR learn* OR play OR psycholog*) AND (develop* OR adjust* OR problem OR function* OR stress OR trauma OR skill* OR resilien*). A total of 13,049 records were identified through database search and imported into EndNote [21]. 9,791 records remained after duplicates were removed manually and were exported into Rayyan [22] for screening and cross-review.

### Screening procedure and selection criteria

**Fig. 1:**
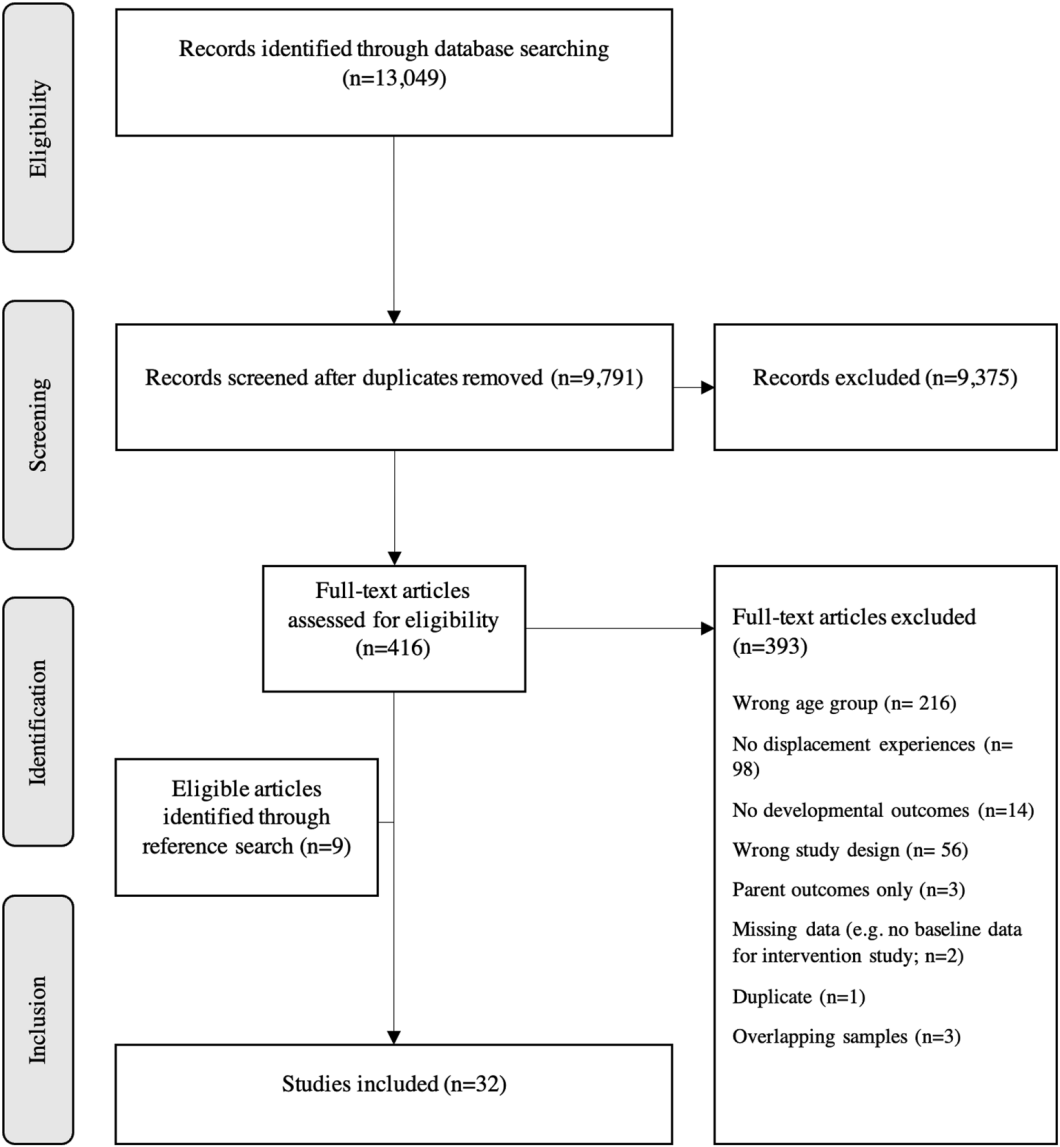
Flow chart of the study selection process

We first screened all search outcomes by title and abstract based on the inclusion criteria. Quantitative studies examining social-emotional and cognitive outcomes in children directly exposed to forced displacement due to political violence (See *Panel 1*) were eligible for inclusion, if results were reported for children aged younger than seven years. Qualitative studies were excluded, as our study group is currently working on a separate review with a specific qualitative focus. Moreover, book chapters, case reports, systematic reviews, study protocols, and theses were excluded. Only pre-intervention data of intervention studies were included. We selected 416 publications for full-text review. Of those, 393 studies were rejected due to the lack of inclusion criteria. We additionally added publications from reference lists and citing literature of included works and authors. In total, 32 publications were included in the review. Figure 1 offers a detailed description of the search and selection process.

### Quality assessment

Risk of bias was evaluated based on the Quality Appraisal Checklist for Correlation and Intervention studies (NICE) [23]. The checklist was applied prior to a final rating of a study’s internal and external validity and was undertaken independently by two authors for each study with a total of six authors (KB, AH, SB, JU, MK, MF). Any discrepant ratings were resolved through group discussion. See Additional file 1 for description of the quality assessment.

### Synthesis

**Fig. 2:**
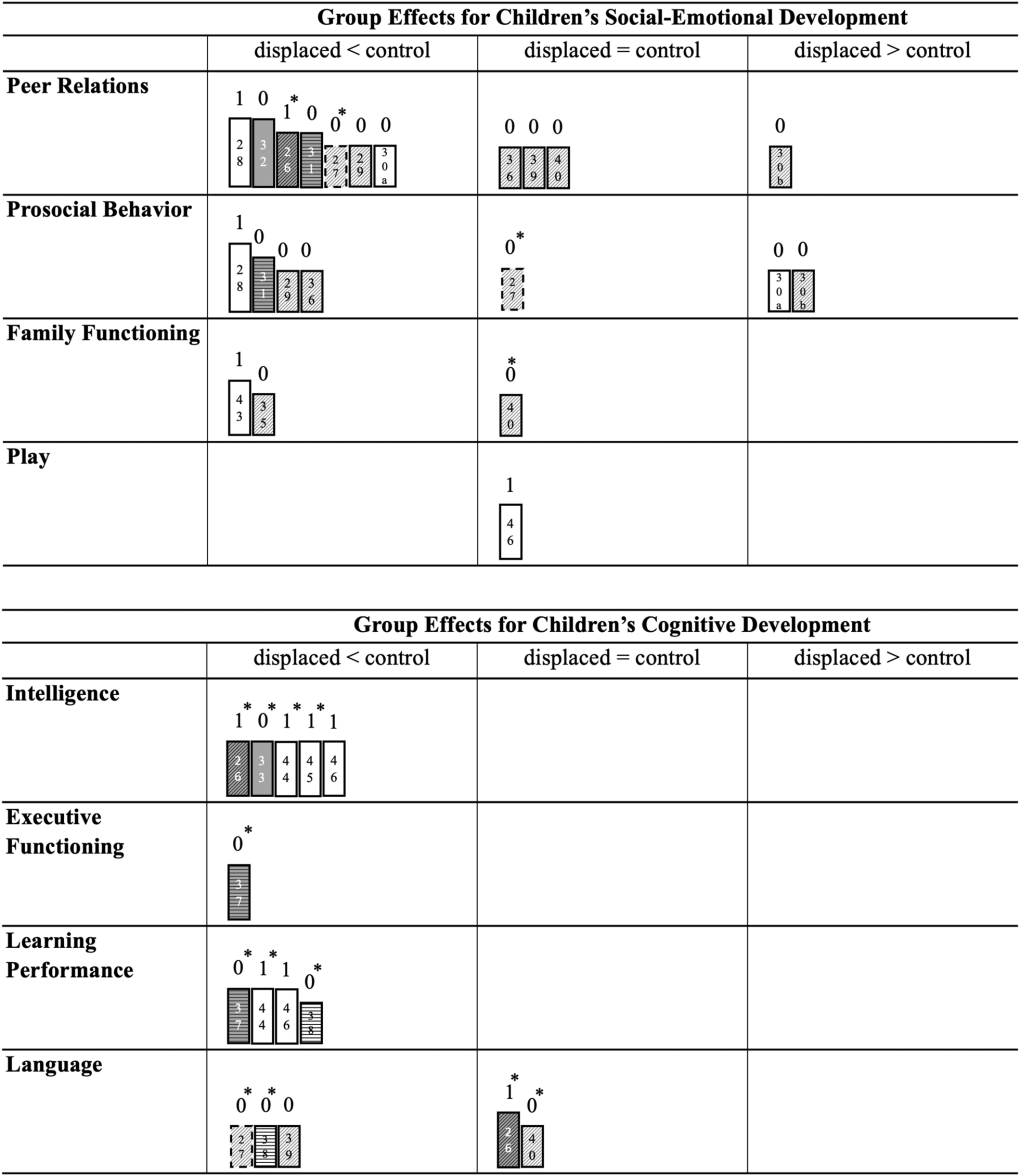
Harvest plots for developmental outcomes. Each study finding is represented in the respective row (outcome variable) and column (direction of group effect) using bars, with height indicating settlement status (highest = detention, middle = refugee camp, small = resettled, dashed = not reported), color denoting number of ACE exposure (white = one ACE category, grey = multiple ACE categories), bar hitching showing time since arrival (full tone =  < 2 years, hitched =  > 2 years, vertical lines = not reported), and the number above the bar indicating control group type (0 = healthy/norm, 1 = clinical/displaced). A * indicates use of parent reports only. References are denoted by numbers within the bars. For follow-up studies, 'a' signifies the initial assessment, and 'b' signifies the follow-up results

Due to the significant methodological heterogeneity among the included studies, evidence was synthesized narratively, comprising the discussion section of this paper. Harvest plots [24] were used to graphically display the distribution of reported group comparisons between displaced and control samples in the outcome domains. Separate matrices were computed for each of the outcome categories consisting of rows (outcome variables) and columns (directions of group effect). Each study finding was represented with a bar that was assigned to the column and row for which that study had reported relevant results and each bar was customized to portray different study and sample characteristics (Fig. 2).

### Adverse childhood experiences

According to the World Health Organization, “Adverse Childhood Experiences (ACE) refer to some of the most intensive and frequently occurring sources of stress that children may suffer early in life” [25], such as abuse, neglect, household dysfunction, and peer, community or collective violence. Based on previous research [6], we adapted this definition of ACEs to different experiences displaced children might encounter, including the following categories: (a) constant of violence; witnessing or experiencing of any type of war or armed conflict (b) witnessing or experiencing the death or injury of a parent or relative, or being separated from family members (c) threat of violence; witnessing, or experiencing violence while in transit (d) exposure to harmful refugee conditions (e.g., immigration detention). As all children included in this review experienced forced displacement, it is not listed as an independent category.

## Results

### Descriptions of included populations

#### Overview

Table 1 shows the key characteristics of displaced samples.

**Table 1:** Study and sample characteristics

Reference	Study population	Housing	N	Age in years M(SD), range	ACE category	ACE exposure n(%)	Time in host country in months M(SD), range	Domains assessed	Measures
26	Internally displaced children and orphans in Ethiopia	refugee camp, orphanage	148	5.7(0.9), 4–7	a,b,d*	a: 148(100), b: 74(50), d: 148(100)	32.4	Peer relations, Intelligence, Language development	Behavioral Screening Questionnaires (BSQ; (Richman, Stevenson & Graham, 1975); The Leiter International Intelligence Scale (Leiter, 1969); The Raven Progressive Matrices (Raven, 1958); Short version of the Token test (McNeal & Prescott, 1978)
27	Refugee children from Middle East, Southeastern Europe, North Africa, and Sub-Saharan Africa in Germany	nr	207	T_1_: 5.78(0.85), 3–7 T_2_: 5.87(0.87) T_1m_: 6.54(0.42) T_c_: 7.18(0.63)	a*	nr	T_1_: 26.4(21.1) T_2_: 26.3(20.0) T_1m_: 27.19(20.75) T_c_: 23.97(16.2)	Peer relations, Cognitive development, Language development	Peabody Picture Vocabulary Test (Dunn & Dunn, 2007); Subtest “Object Assembly” from the Wechsler Preschool and Primary Scale of Intelligence-III (Wechsler, 2002); Intelligence and Development Scales (Grob et al., 2009); Strength and Difficulties Questionnaire (SDQ; Goodman, 1997)
28	Refugee children from Eastern Mediterranean, Southeast Asia, Western Pacific, Africa in Sweden	detention	86	8.4, 4–15	d	48(55)	7.27	Peer relations, Prosocial behavior	Strengths and Difficulties Questionnaire (SDQ; Goodman, 2001)
29	Refugee children in Germany	nr	study 1: 84 study 2: 50 study 3: 107	study 1: 3·86(1.18) study 2: 3·92(1.22) study 3: 5.79(0.85)	a*	nr	study 1: nr study 2: 43.87(37.59) study 3: 25.4(20.11)	Peer relations, Prosocial behavior	Child-Teacher Report form 1·5–5 (C-TRF; Achenbach & Rescorla, 2000); Strengths and Difficulties Questionnaire (SDQ; Goodman, 2001)
30	Refugee children from Southeast Asia, Africa, Eastern Mediterranean in Sweden	settled	61	6.0, 05–15	d	nr	baseline: 13 follow-up: 31	Peer relations, Prosocial behavior	Strengths and Difficulties Questionnaire (SDQ; Goodman, 2001)
31	Refugee children from Myanmar in Bangladesh	refugee camp	622	0-to 2 year old’s: 0.86(0.54), 2-to 16 year old’s: 6.81(3.53)	a,d*	nr	nr	Peer relations, Prosocial behavior, Cognitive development, Language development	Developmental Screening Questionnaire (DSQ: Khan et al., 2013); General development assessment (GDA); Strengths and Difficulties Questionnaire (SDQ; Goodman, 2001)
32	Refugee children from South America in the U.S	settled	42	6.79(4.42), 2–16	a,c*	nr	1·17	Peer relations	Strengths and Difficulties Questionnaire (SDQ; Goodman, 2001)
33	Internally displaced children in Bosnia	refugee camp, private homes	87	5.5, 5–6	a,b,d	nr	nr	Intelligence	War Trauma Questionnaire (Macksoud, 1992); Raven’s Colored Progressive Matrices (CPM; Raven, 1947); Birleson’s Depression Inventory (BDI; Birleson, 1981); Child Assessment
34	Refugee children in Sweden	settled	43	6·0, 0.5–15	d	nr	baseline: 13 follow-up: 31	Peer relations, Prosocial behavior	Strengths and Difficulties Questionnaire (SDQ; Goodman, 2001)
35	Internally displaced children in Colombia	settled	279	4.2(1.0), 1.5–5	a	nr	12–60	Family functioning	The General Functioning (GF) Scale of the Family Assessment Device (FAD; Byles et al., 1988); KiddieSADS PTSD Traumatic Event Checklist (Kaufman et al., 1996)
36	Refugee children from Syria in Turkey	settled	40	5.57, 5–6	a*	18(90)	12–36	Peer relations, Prosocial behavior	The Ladd-Profile Child Behavior Scale (Ladd & Profilet, 1996); Peer Victimization Scale (Kochenderfer & Ladd, 2002)
37	Refugee children from Syria (S) and internally displaced children in Iraq (Y)	refugee camp	150	Y: 5.92(0.55), S: 5.73(0.58)	a,d*	nr	nr	Executive functioning, Math abilities, Short term memory	Backward and Forward Word Span Task (Lanfranchi et al., 2004); Stroop Task (Gerstadt et al., 1994); Numerical Intelligence Battery (BIN; Molin et al., 2007)
38	Refugee Children from Syria in Turkey	settled	373	5.11(0.48), 5–6	a*	nr	nr	Peer relations, Language development, math abilities,	Preliteracy and Prenumeracy Skills Scale (Adato & Bekman, 1989); Turkish Early Language Development Test (TEDIL-3; Güven & Topbas, 2014); Emotion Regulation Checklist (Shields & Cicchetti, 1997); Social Competence and Behavioral Assessment Scale (SCBAS; LaFreniere & Dumas, 1996)
39	Syrian refugee children in Turkey	settled	120	1.5–6	a	43.3%	90% longer than 1 year	Peer relations, Language Development	Denver II Developmental Screening Test (DDST-II; Frankenburg et al., 1989)
40	Refugee children from Syria in Turkey	settled	70	5.56(1.09), 0–6	a	0(0)	36	Family functioning, Cognitive development, Language development	The Diagnostic Classification of Mental Health and Developmental Disorders of Infancy and Early Childhood: Revised Edition (DC:0–5:Zeanah et al., 2016)
41	Refugee children from Syria and Iraq in Germany	nr	42	283(1.54), 0.42–5.58	a	24(60)	21.53(11.57), 1–40	Family functioning	Parenting Interactions with Children Checklist of Observations Linked to Outcomes (PICCOLO; Roggman et al., 2013)
42	Internally displaced children in Israel	returned to homes	107	3.9	a	107(100)	30	Family Function	Child Behaviour Checklist 1·5–5 years (CBCL 1·5–5; Achenbach & Edelbrock, 1983); Preschool Children's Assessment of Stress Scale (PCASS; Laor et al., 1996); Change of Functioning Scale (CFS; Laor et al., 1996); Vineland Adaptive Behavior Scales (Sparrow et al., 1984)
43	Internally displaced children in Israel	hotels	230	4.0, 3–5	a	230(100)	6	Peer relations, Family function	Child Behaviour Checklist 1·5–5 years (CBCL 1·5–5; Achenbach & Edelbrock, 1983); Preschool Children's Assessment of Stress Scale (PCASS; Laor et al., 1996); Change of Functioning Scale (CFS; Laor et al., 1996); Childhood Personality Scales (Cohen et al., 1977); Family Adaptability and Cohesion Evaluation Scales (FACES; Olson, 1986)
44	Refugee children in Germany	refugee camp	109	5.10(1.15), 3–7	a	73(67)	16.5	Intelligence, Short term memory	Child and Adolescent Trauma Screening (CATS; Sachser et al., 2016); Scale of Intellectual Functioning (SIF) of the Kaufmann-Assessment-Battery for Children (KABC-II; Kaufmann et al., 2015)
45	Refugee children mostly from Nigeria, Afghanistan, and Syria in Germany	refugee camp	72	5.14(1.17), 3–7	a	32(61·5)	19(17.72), 0.5–60	Peer relations, Prosocial behavior, Intelligence, Short term memory	Child and Adolescent Trauma Screening (CATS; Sachser et al., 2016); Strengths and Difficulties Questionnaire (SDQ; Goodman, 2001); TST assessment form (Saxe et al., 2016); Scale of Intellectual Functioning (SIF) of the Kaufmann-Assessment-Battery for Children (KABC-II; Kaufmann et al., 2015)
46	Refugee children in Germany	refugee camp	181	4.94 (4.66 5.22)	a	62(89)	3.71	Social-emotional competencies, Play, Intelligence, Learning Performance	Play Observation Scale (Bernhardt & Hahnefeld, 2021); Behavior Observation Scale for Preschool Children (Verhaltensbeurteilungsbogen für Vorschulkinder; VBV-ER 3–6; Döpfner, 1993); Scale of Intellectual Functioning (SIF) of the Kaufmann-Assessment-Battery for Children (KABC-II; Kaufmann et al., 2015)
47	Refugee children from Asia and Africa in Australia	detention	110	6.0(4.72)	a,b,d	a: nr b: nr d: 605(97.3)	13	Cognitive development	Centers for Disease Control and Prevention/ Adverse Childhood Experiences (CDC/ACE; Hanes, 2017)
48	Refugee children from Iran, Iraq, Afghanistan, and Palestine in Australia	detention	20	0.92–17	a	20(100)	15, 12–18	Family functioning, Play	Child Assessment
49	Refugee children from Iraq, Kosovo, Pakistan, Russia, Somalia, Sri Lanka, and Sudan in Belgium/ Denmark	nr	18	6.7(2.2), 4–9	a	8(44.4)	nr	Family functioning	Attachment Story Completion Task (ASCT; Verhueren et al., 1996)
50	Refugee children from Syria in Germany	refugee camp	96	7.2, 0–14	a,b,c	a: 96(100)	1.3	Language development	Parent Interview; Child Assessment; Post-traumatic Stress Disorder Semi-Structured Interview (PTSDSSI; Scheeringa & Zeanah, 1994)
51	Refugee children in Germany	refugee camp	2551	5.72	nr	nr	28.43	Cognitive reasoning, Language development	Peabody Picture Vocabulary Test (PPVT-4; Dunn & Dunn, 2007); NEPS-MAT (Lang et al., 2014)
52	Internally displaced children in Israel	refugee camp	study 1: 74 study 2: 191	4.7(1.34), 2–7	a	264(99.6)	0.53(0.26)	Play	War Related Experiences Scale (Sadeh et al., 2008); Stress Reaction Checklist (SRCL; Sadeh et al., 2008)
53	Refugee children from Iran in Sweden	settled	50	8.4, 4–8	a	42(84)	42	Peer relations, Language development	Parental Interview; Child Assessment; Erica Method/World of Technique (Lowenfeld, 1950)
54	Refugee children from Iran in Sweden	settled	39	8.4, 4–8	a	34(87)	baseline: 12 follow-up: 42	Play	Parental Interview; Erica Method / World of Technique (Lowenfeld, 1950)
55	Refugee children from Iran in Sweden	settled	50	5.83, 4–8	a,b	42(84)	nr	Play	Parental Interview; Erica Method / World of Technique (Lowenfeld, 1950)
56	Refugee children from former Yugoslavia in Sweden	refugee camp	66	5–15	a	24 (37)	5	Family functioning, Play	Parent Interview
57	Refugee children from Middle East and Africa in Sweden	70% settled	61	3.75(1.4), 2–6	nr	9(15)	nr	Peer relations, Prosocial behavior	Strengths and Difficulties Questionnaire (SDQ; Goodman, 2001); Primary Care PTSD Screen (PC-PTSDM Prins et al., 2003); Child PTSD Symptom Scale (CPSS; Nixon et al., 2013)

ACE categories

a constant of violence; witnessing or experiencing of any type of war or armed conflict

b witnessing or experiencing the death or injury of a parent or relative, or being separated from family members

c threat of violence; witnessing, or experiencing violence while in transit

d exposure to harmful refugee conditions (e.g., immigration detention)

PTSD posttraumatic stress disorder

^*^Description by the authors only

A total of 6,878 children aged 0- to 17-years were included in the reviewed studies, of which 5,858 were younger than seven years. Included studies were published between 1993 and 2023. Twenty-one studies conducted comparisons analyses with displaced populations [26–29], norm populations [27–34], healthy controls [35–39], children displaced in second generation [40, 41], and non-displaced children from war-zones [42, 43], and in clinical settings [29, 44–46]. Fourteen studies had at least partly overlapping samples. We included those studies if distinct outcomes were reported or contacted the authors to make separate calculations, if possible.

#### Flight history

Studies were conducted in Australia [28, 30, 34, 47, 48], Bangladesh [31], Bosnia-Herzegovina [33], Colombia [35], Denmark/Belgium [49], Eritrea [26], Germany [27, 29, 41, 44–46, 50, 51], Iraq [37], Israel [42, 43, 52], Sweden [53–57], Turkey [36, 38–40], and the US [32] and included children from the Middle East (Iraq, Syria, Afghanistan, Iran, Israel, Palestine, Pakistan, Lebanon), Africa (Somalia, Tunisia, Nigeria, Eritrea, Sudan), Asia (Myanmar, Sri Lanka), Central and South America (El Salvador, Honduras, Guatemala, Chile, Columbia), the Western Pacific, and Eastern Europe (Russia, Former Yugoslavia). Four studies included unaccompanied children [26, 31, 47, 48].

### Quality assessment

As for internal validity, three (9.3%) studies met the criteria for high quality, 14 (43.8%) for moderate quality and nine (28.1%) studies were of low quality, mostly due to not controlling for confounding factors, solely relying on parents as informants or using insufficient analytical methods. Quality of external validity was rated as good in six (18.8%) studies, as moderate in 22 (68.8%) studies and as low in four (12.5%) studies, due to restricted generalizability of study findings (See Additional file 1). With respect to the methodical and practical challenges that can arise when conducting research with displaced populations [8, 45, 46, 58, 59], no studies were excluded due to low quality rating.

### Outcomes

Categorization of the outcome variables employed is detailed in Table 2. Information sources included parent and caregiver reports, medical records, and child assessment by investigators. Nineteen studies used a multiple source approach [26, 27, 29, 33, 38, 40, 41, 44–51, 53–56] and in eleven studies, parent reports were the only source of information [28, 30–32, 34, 35, 42, 43, 52, 56, 57]. Modes of data collection were questionnaires [26–36, 38, 41–46, 50, 52, 57], structured interviews [40, 48, 51, 53, 54, 56], clinical observations [26, 27, 33, 37–41, 44–46, 48–51, 53–55], and review of mental health records [47].

**Table 2:** Outcome categories in reviewed studies

Category	Outcome	N of articles (%)
Socio-emotional outcomes	Peer relations	14 (43.8%)
	Prosocial behavior	6 (18.8%)
	Family functioning	7 (21.9%)
	Play	7 (21.9%)
Cognitive outcomes	Intelligence	7 (21.9%)
	Learning performance	5 (15.6%)
	Language	11 (34.4%)

#### Social-emotional outcomes

Experience of forced displacement was associated with difficulties in young children’s peer relations (21–57.1%) [26–29, 31, 32, 40, 53, 54], such as not having friends to play with (15.6%) [31, 53], being offensive towards peers [26], and being bullied by peers [53], while six studies did not find group differences regarding displaced children’s social-emotional competencies [34, 36, 38, 39, 42, 46]. Four studies described less prosocial behavior in displaced children compared to controls (10.3%) [28, 29, 31, 36], while one study reported the opposite effect [30], and one study found no significant group differences in this domain [27]. Six studies pointed out the co-occurrence of displacement experiences, disrupted family dynamics and children’s symptomatology [35, 40, 41, 43, 48, 49]. Poor family function, overdependency on caregivers and separation fears were prevalent [52, 54], and significantly more frequent in displaced than non-displaced participants in two studies [35, 43], while one study reported comparably high prevalence of attachment disturbances in children with direct and family background of displacement [40]. Child-caregiver relationships were reported to be characterized by avoidant attachment, parental absence, low maternal affection, and oppositional behavior [41, 48, 49], which was further correlated with children’s symptom load [35, 43]. Exposure to forced displacement was associated with disturbed play in 6–62% of investigated children, reflected as reenacting, repetitive or unstructured play or general disinterest or passivity in play activities [48, 52, 54–56]. However, one study did not observe reenacting and emotionless-cold play patterns among refugee children [46]. In the same study, refugee children showed comparable play development, but less social interaction during play compared to a clinical comparison group.

#### Cognitive outcomes

Overall, 23–78.5% of displaced children were reported to perform low on cognitive measures [26, 27, 30, 31, 33, 37, 40, 44, 45, 47]. Three studies in German refugee camps found displaced children’s nonverbal IQ scores to be normally distributed, with means more than one standard deviation below those of the German norm population [44–46]. Dybdahl [33] reported IQ scores of children exposed to the Bosnian war on 25th percentile for European and US norms. Wolff et al. [26] reported 6- to 7-year-old, displaced orphans to perform better on cognitive and language measures than accompanied displaced children. However, the authors did not report exact IQ scores for both groups. Further, learning performance [37, 44, 46], executive function [37], and early math abilities [37, 38] were all reported to be less developed in displaced children compared to control groups. Eleven studies detected limited speech capacities in displaced children (7–50%) [26, 27, 30, 31, 38–40, 48, 50, 51, 53], that persisted up to 3 ½ years after settlement [30, 53].

### Influencing factors

Twenty-seven (84.4%) of the included studies conducted correlational, regression, or group analyses to identify influencing factors of children’s developmental outcomes after forced displacement.

#### ACEs

Exposure to different forms of adversity was positively associated with separation fears [43], play behavior [46, 53, 55, 56], language and social-emotional development [39], and was among the factors that most strongly determined children’s social adjustment at follow-up [53]. As can be seen in our Harvest plots, all but one studies investigating displaced children exposed to cumulative ACEs reported higher levels of disturbance compared to control groups. Separation from one or both parents thereby emerged as one of the most important risk factors for social-emotional and cognitive developmental problems [26, 31, 33, 34, 55]. Displaced children from war and non-war zones did not differ in regard to peer problems in one study [29], and four studies did not relate flight duration to learning performance, non-verbal IQ, family functioning and play behavior [41, 44–46].

#### Settlement

Follow-up studies suggested a decrease in developmental concerns within the first 3- to 4-years of settlement [30, 36, 42, 43, 53, 54], while children’s social adjustment improved over time [53]. Harvest plots accordingly indicate that children who stayed in the host country for shorter periods of time were more symptomatic than control children. While time in the host country was associated with better language and learning performance [27, 44, 51], and social interaction during play [46], nine studies did not link time since arrival in the host country and residence status with children’s developmental state [26, 28, 29, 34, 35, 44, 51, 55], and family functioning [48]. Four studies found that (longer) attendance of childcare centers was correlated with improvement on measures of peer problems, prosocial behavior, nonverbal reasoning and language abilities [27, 29, 38, 51], although effects did not apply to cognitive development [27] and play behavior [46] in two studies. Four studies reported comparable outcomes for children in the first and second generation of displacement [34, 35, 40, 41]. Accordingly, the impact of post-migration stressors, such as unstable housing, on children’s development was highlighted across studies [26, 29, 34, 42–44, 56]. Eight out of the nine studies conducting group comparisons with children living in refugee and detention centers reported developmental levels below norm, indicating that placement in refugee accommodations jeopardizes social-emotional and cognitive functioning, with risk increasing the longer children are living in such environments [48, 56]. Children held in immigration detention, especially, were shown to be at higher risk than non-detained displaced children with similar exposure to pre-arrival adversity [28] and displayed the highest prevalence of developmental concerns across all included groups in this review [28, 32, 47, 48].

#### Children’s age and gender

Twelve studies analyzed the effect of age and gender on children’s reactions to forced displacement. In five studies, the youngest age groups showed relatively more social-emotional problems [42, 43] and reenactments in play [55], and performed less advanced than older peers on cognitive and language measures [26, 27]. Moreover, one study found age effects for play development in clinical comparison, but not refugee children [46]. Findings on the effects of gender were mixed and pointed towards increased vulnerability for both boys [53–55] and girls [33], who showed more social play and prosocial behavior in two studies [27, 46]. Six studies did not report any effects of age or gender on presented outcomes [26, 33, 34, 41, 43, 50].

#### Parental factors

Parental distress, mostly mother’s symptoms, strongly affected displaced children’s adaption in the host country, socialization and play behavior in children [42, 43, 46, 53], while fathers mental health was only assessed in one study [53]. Limited maternal affection thereby was reported as risk factor for children’s development [41], while increased affection towards the child [41] and optimism of mothers [56] were reported as potential protective factors.

## Discussion

In addition to previous findings reporting high prevalence of the categorical diagnosis of PTSD among displaced children [19], this review was novel in its developmental focus and showed that young children’s social-emotional, cognitive and language development is negatively affected by displacement experiences. Solely focusing on mental health outcomes by applying the categorical criteria for PTSD does not sufficiently reflect the diversity and interplay of possible reactions in young infants, that might differ from expressions of older children and adults [60].

The wide-ranging prevalence rates for developmental issues presented in this review indicate that children might not only be impacted by the experience of displacement itself, but also by associated stressors. Forced displacement in and of itself is not only an adverse experience, but also increases the likelihood of experiencing further ACEs and structural inequalities [6]. Despite lacking detailed descriptions of experienced ACEs, the included studies suggest that risk factors for poor outcomes include cumulative exposure to war-experiences, prolonged stay in immigration centers, family separation and parental distress. This emphasizes the role of contextual variables during and after flight rather than restricting investigations to the direct effects of pre-displacement and flight events.

The impact of family separation on displaced children’s outcomes highlights their dependency on parental support [26, 31, 33, 55], particularly for the youngest age groups [35, 42]. The family environment can either form a protective shield against adverse experiences or jeopardize children’s development, if parents are themselves distressed and struggle to respond to children needs in a sensitive way, crucial to engage in developmental tasks [61]. Indeed, parental distress and loss of family function were identified as important risk-factors for children’s development in several studies included in this review [41–43, 46, 53, 56]. As it is often also structural inequalities that make it difficult for parents to give their children a sense of security and stability, parents should be supported in creating an atmosphere of normality for their children by maintaining daily routines and small rituals even under difficult conditions.

All studies conducted with children living in refugee and detention centers reported poor outcomes in at least one of the outcome domains, pointing to the negative consequences of prolonged stays in transitory settings [28]. Time since stable resettlement on the other hand emerged as a protective factor influencing children’s development in a positive way [30, 36, 42, 43, 53, 54]. Although the exact circumstances of resettlement were not described in most of the included studies, it can still be assumed that as families resettle in the host country, their environment is likely to become more stable, as children gain access to health care and formal education. Thus, the effect of displacement is likely to be moderated by the context in which time is spent. The protective effect of early childcare and preschool education has been emphasized across studies included in this review [26–28, 34, 38, 44] and in recent literature [12, 13, 17, 62, 63]. Daycare centers can provide displaced children with a child-friendly and playful learning environment known to promote social-emotional and cognitive development, which can give them a sense of stability, security and belonging that might reduce symptoms of distress [27, 38]. At the same time those factors have been shown to facilitate transitions to school systems and promote academic learning and cohesion between displaced and local children [38], as preschool settings offer opportunities for displaced children to catch up in development with their peers. On the other hand, unresolved asylum claims often limit access to preschool institutions. Therefore, an important opportunity is lost to improve chances for positive developmental trajectories and integration in the long-term [34].

### Limitations

This systematic review was limited by the quality of included studies, of which 25 were cross-sectional and 14 were given a low rating regarding their internal and/or external validity. The large heterogeneity across studies did not allow for a statistical analysis conducted in meta-analyses. While the resulting diversity among studies may restrict their comparability, it allowed for the creation of a more complex picture of the effects on diverse displaced populations in different settings.

Assessing young children presents a challenge as they may lack the ability to comprehend or verbalize their experiences, making it necessary to rely on information provided by the parents. The validity of parental reports has been discussed in previous literature, as the perception of their children’s well-being and development might be influenced by their own symptomatology [45, 46]. Eleven studies used parents as their only data source which may have resulted in over- or underestimation of children’s development. Further, the use of standardized measures that may not have been culturally appropriate questions the validity of reported findings [44, 64]. Confronting displaced populations with measures normed for Western contexts presupposes that understandings of psychological, behavioral or developmental phenomena and manifestations of distress can be generalized across different cultures [64]. Developmental tests commonly used in study settings are usually created and normed for children who already know comparable play material and assignments from educational contexts, disadvantaging displaced children without former educational experience [44]. This is especially critical, when measures are not only utilized for research purposes, but in educational or clinical settings to determine school suitability. As most children included in this review did not attend any kind of childcare or educational facilities, the reported developmental outcomes could therefore be interpreted as limited test performance rather than developmental impairments [26, 29].

Only ten (31.3%) of the reviewed studies were conducted in middle- or low-income countries, even though 83% of refugees are hosted in developing countries [10]. Resources and support services are especially restricted in those settings and therefore contribute to the vulnerability of young children placed within them [38].

### Implications

#### Further research

Longitudinal assessments of age-specific developmental outcomes are needed to improve our understanding of the diverse reactions in young, displaced children—in high- but also in middle- or low- income countries. Investigation of prewar levels or comparisons with peers from the home country can elucidate whether test materials are biased towards children with experience in Western educational settings. Future researchers should make use of culturally appropriate assessment tools and incorporate modes of nonverbal expressions of children and educator reports as a supplement to parent rating.

Current empirical research often uses approaches that classify developmental outcomes in displaced persons as pathologic rather than seeing it as an expected response to facing the various challenges associated with forced displacement [18, 65, 66], therefore underestimating their ability to reach their full developmental potential when provided with the necessary support systems and stable living conditions [67]. Although this article highlighted the negative effects of displacement experiences, several results point towards the resilience of young children. Overlooking the strengths of displaced populations perpetuates a deficit view and places them at risk for discrimination, which has been shown to directly affect psychological distress in youth and adolescent refugees [68]. Instead, there is a need for studies that draw on children’s reactions to forced displacement from a resilience perspective in order to understand how both individual qualities, social relations, and modifiable contextual factors contribute to children’s acculturation, well-being and positive developmental trajectories in the long-term [17, 18].

Displaced children are especially vulnerable to high risks for ACEs, emphasizing the need of systematic assessment of those experiences to develop effective intervention strategies. Common definitions of ACEs focus on adversities children face within the family, while political violence and forced displacement are currently not enough covered by the framework [6, 69, 70]. It is therefore necessary to develop a broader definition that reflects the experiences that displaced children face outside the household.

Our review encompasses studies on both refugee and internally displaced children. While studies on internally displaced children did not provide prevalence data, they indicated challenges in peer relations, family functioning, and cognitive and language development [26, 33, 35, 42, 43, 52], similar to findings for refugee children. Despite expectations of higher difficulties for refugee children due to prolonged flight durations and acculturation challenges, internally displaced children faced unique adversities—residing in shelters, orphanages, or close to conflict zones, exposing them to repeated war and violence experiences. Most studies with internally displaced children were conducted in Israel where the initiating conflict was unresolved, which has been recognized as risk factor for internally displaced persons mental health [71]. Additionally, post-displacement stressors, such as unstable housing and unemployment have been reported for internally displaced populations [72], and further underscore the vulnerability of these children. Notably, no studies have explored distinctions between these two populations, representing a compelling avenue for future research.

#### Political implications

This review shows that the negative effects of forced displacement on children, even at a very young age, are wide-ranging and powerful; however, stressors experienced in the host country, though prevalent, are modifiable. Post-migration factors provide opportunities for governments to contribute to secure and stable environments for displaced populations by keeping exposure to camps and reception centers at a minimum, preventing family separations and guaranteeing quick access to healthcare and educational institutions. Especially in low-resource environments, participation in socially inclusive preschool programs can promote healthy development and school readiness [27, 38]. Young, displaced children are vulnerable, especially because of their dependency on caregivers, who might have also undergone stressful and traumatic experiences [8]. Interventions should offer practical and mental health support for caregivers and provide childcare and education programs to shield children from post-displacement adversity. Ideally such resources should be available from the time of arrival. A resilience-based research approach is essential which instead of focusing on an individual's ability to cope with extreme situations draws on social and environmental factors and political responsibilities to protect and provide vulnerable populations with resources and support systems [38].

## Conclusion

Although existing research with displaced children is limited, particularly regarding young children and children in low- or middle-income settings, there is no doubt that forced displacement and associated ACEs have negative effects on children’s development. Reactions of 0- to 6-year-old children are diverse and crucially influenced by contextual factors such as housing situation, separation from family members or parental distress. Our findings reinforce the importance of creating policies and practices that provide access to healthcare and early education and support the stable settlement of these children and their families to promote resilience and positive developmental and integration trajectories in young, displaced children.

**Panel 1:** Definitions.

Refugee: A person who is, due to fear of being persecuted because of their belonging to a certain race, religion, nationality, social group or political opinion, outside the country of his nationality and is unable or, owing to such fear, is unwilling to return to it [73].

Internally displaced person: A person who has been forcibly displaced from their home or place of residence as a result of or in order to avoid the effects of armed conflict, situations of generalized violence, violations of human rights or natural or human-made disasters, and who has not crossed an internationally recognized state border [74].

Forcibly displaced person: A person who was forced to flee their home due to conflicts, violence, fear of persecution or human rights violations, including internally displaced persons, refugees, asylum seekers and Venezuelans displaced abroad [75].

Political violence: Violence perpetrated by state- or non-state representatives to achieve or prevent politically motivated goals. Examples include war, genocide, terrorism, denial of citizenship, wrongful detention, enslavement or forced displacement. Another form of political violence can be non-action of a government such as lack of political representation or discrimination in the provision of civil services for women or other minority groups [76].

### Supplementary Information

**Additional file 1: **Quality appraisal of the included studies based on NICE-criteria.

## Data Availability

The datasets used and/or analysed during the current study are available from the corresponding author on reasonable request.

## Abbreviations

PTSD: Post-traumatic stress disorder
ACE: Adverse childhood experience
IQ: Intelligence quotient
