# Supplementary material for: Young children’s development after forced displacement: a systematic review
Source: Child Adolesc Psychiatry Ment Health. 2024 Feb 1;18:20. doi: 10.1186/s13034-024-00711-5 (PMC10835848; doi:10.1186/s13034-024-00711-5)
Supplement: Supplementary file 1 — Additional file 1: Quality appraisal of the included studies based on NICE-criteria. [file 13034_2024_711_MOESM1_ESM.docx]

**Appendix** Quality appraisal of the included studies based on NICE-criteria

| **Study** | **1.1** | **1.2** | **1.3** | **2.1** | **2.2** | **2.3** | **2.4** | **3.1** | **3.2** | **3.3** | **3.4** | **3.5** | **4.1** | **4.2** | **4.3** | **4.6** | **5.1** | **5.2** |
| --- | --- | --- | --- | --- | --- | --- | --- | --- | --- | --- | --- | --- | --- | --- | --- | --- | --- | --- |
| 26 | ++ | ++ | ++ | NA | + | NA | + | + | + | + | NA | NA | NR | + | ++ | + | ++ | + |
| 27 | ++ | ++ | + | + | + | ++ | - | + | + | + | NA | + | NR | + | ++ | ++ | ++ | + |
| 28 | ++ | + | + | NA | + | NA | - | - | + | - | NA | NA | NR | - | ++ | + | - | + |
| 29 | ++ | ++ | + | NA | + | NA | - | + | + | + | NA | NA | NR | + | ++ | + | + | ++ |
| 30 | ++ | + | + | NA | + | NA | - | - | + | - | NA | NA | NR | + | ++ | + | - | ++ |
| 31 | ++ | ++ | ++ | NA | + | NA | - | + | + | - | NA | NA | NR | + | + | + | + | ++ |
| 32 | ++ | ++ | ++ | NA | + | NA | + | - | ++ | + | NA | NA | NR | + | + | + | - | ++ |
| 33 | ++ | + | + | ++ | + | + | + | + | + | ++ | ++ | ++ | NR | ++ | + | + | + | + |
| 34 | ++ | + | + | NA | + | NA | + | - | + | + | NA | NA | NR | + | ++ | + | - | + |
| 35 | ++ | + | + | NA | + | NA | ++ | - | + | ++ | NA | NA | NR | ++ | ++ | ++ | - | + |
| 36 | ++ | ++ | + | NA | + | NA | + | - | + | + | NA | NA | NR | + | + | + | - | + |
| 37 | ++ | ++ | ++ | NA | ++ | NA | + | ++ | + | + | NA | NA | NR | + | ++ | ++ | ++ | + |
| 38 | ++ | + | + | NR | ++ | ++ | - | + | + | + | ++ | + | + | + | ++ | ++ | + | + |
| 39 | + | ++ | + | NA | + | NA | + | + | + | ++ | NA | NA | NR | + | + | + | + | + |
| 40 | ++ | + | + | NA | + | NA | - | + | + | + | NA | NA | NR | - | + | + | + | + |
| 41 | ++ | + | + | NA | + | NA | ++ | + | ++ | + | NA | NA | NR | ++ | ++ | ++ | + | + |
| 42 | ++ | + | + | NA | + | NA | ++ | - | + | ++ | NA | NA | NR | ++ | ++ | ++ | - | + |
| 43 | ++ | + | + | NA | + | NA | ++ | - | + | ++ | NA | NA | NR | ++ | ++ | ++ | - | + |
| 44 | ++ | + | + | NA | ++ | NA | ++ | + | ++ | ++ | NA | NA | NR | ++ | ++ | + | + | ++ |
| 45 | ++ | + | + | NA | ++ | NA | ++ | + | ++ | ++ | NA | NA | NR | ++ | ++ | + | + | ++ |
| 46 | ++ | ++ | ++ | NA | + | NA | + | + | ++ | ++ | NA | NA | + | ++ | ++ | + | + | + |
| 47 | ++ | - | - | NA | + | NA | + | NR | - | + | NA | NA | NR | + | - | + | - | - |
| 48 | ++ | - | - | NA | + | NA | - | NR | + | - | NA | NA | NR | - | - | + | + | - |
| 49 | + | - | - | NA | + | NA | - | + | + | - | NA | NA | NR | + | - | + | + | - |
| 50 | ++ | + | + | NA | ++ | NA | + | ++ | + | + | NA | NA | NR | + | ++ | + | + | + |
| 51 | ++ | ++ | ++ | NA | ++ | NA | + | + | + | + | NA | NA | NR | ++ | + | + | + | + |
| 52 | ++ | + | + | + | + | + | + | - | + | + | ++ | + | NR | + | + | + | - | + |
| 53 | ++ | ++ | + | NA | + | NA | ++ | + | + | ++ | NA | NA | NR | ++ | + | + | + | + |
| 54 | ++ | ++ | + | NA | + | NA | + | + | + | + | NA | NA | NR | + | - | - | + | + |
| 55 | ++ | ++ | + | NA | + | NA | + | + | + | + | NA | NA | NR | + | - | - | + | + |
| 56 | ++ | + | + | NA | ++ | NA | + | - | + | + | NA | NA | NR | ++ | - | + | - | - |
| 57 | + | + | + | NA | + | NA | + | - | + | + | NA | NA | NR | + | ++ | + | - | + |

++ = good quality, + = moderate quality, - = low quality, NR = not reported, NA = not applicable. The criteria concerning selection of exposure and control groups as well as possible contaminating factors of the intervention and follow-up time for control and exposure groups were not applicable for most studies as they were designed to evaluate the quality of intervention studies. Since the methodology and applicability of interventions was not relevant to our research question, these criteria were omitted when rating the overall internal validity. The settings of refugee centers as well as asylum procedures varied across the different studies and countries and could not always be traced. Thus, no comparability with UK standards could be established and was chosen to not be significant to our research question. The rating of external validity of studies was also assessed with respect to this issue, as generalizability of the published findings may be restricted due to the specificity of the research samples and settings.
